# Emergent Nonlinearity in Active Molecular Chemotaxis

**DOI:** 10.1021/acsnano.6c03464

**Published:** 2026-05-13

**Authors:** Xiaotian Lu, Ayusman Sen, R. Dean Astumian

**Affiliations:** † Department of Chemical Engineering, 8082 The Pennsylvania State University, University Park, State College, Pennsylvania 16802, United States; ‡ Department of Chemistry, The Pennsylvania State University, University Park, State College, Pennsylvania 16802, United States; § Department of Physics and Astronomy, 6250 University of Maine, Orono, Maine 04469, United States

**Keywords:** active matter, enzyme chemotaxis, kinetic asymmetry, chemotactic velocity, nonequilibrium steady state

## Abstract

Understanding chemotaxis at the molecular level is challenging, as individual enzyme molecules cannot sense chemical gradients across their nanometer-sized bodies. Typical theoretical models encompass chemotaxis under constant, externally imposed gradients; however, this overlooks a critical feedback loop, where the active enzymes themselves reshape the imposed gradients through catalysis. In this work, we investigate the principles of active molecular chemotaxis using a Fokker–Planck model for an ATP-driven kinase-phosphatase system. Using experimentally relevant enzyme concentration ranges (∼nM), we demonstrate that the chemotactic velocity of enzymes does not simply respond linearly to chemical gradients, as commonly observed in microscale systems driven by diffusiophoresis. Instead, it emerges from a nonlinear coupling between the enzyme’s spatial distribution, its conformational state (free/bound-state ratio), and chemical gradients modulated by catalytic reactions. As a result, the spatial profile of chemotactic velocity transitions between monotonic and nonmonotonic regimes, depending on substrate availability. Furthermore, we find that high catalyst concentrations can amplify the effective interaction between enzymes, forming a cascade that is critical for collective assemblies such as metabolon formation. To understand these complex interactions, we construct chemotactic velocity maps as a function of enzyme concentration, energy, and substrate availability, offering a set of design principles. This work clarifies the distinct roles of energy, gradients, and enzyme free/bound states in molecular motion, highlighting a fundamental difference between nano and microscale systems, and provides a theoretical framework for designing advanced autonomous active molecular systems.

## Graphical abstract

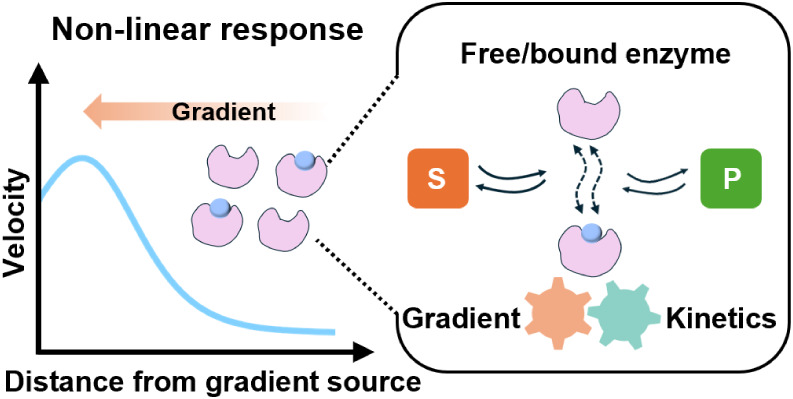

## Introduction

Active matter can use scalar energy to generate higher-order vectorial outputs (chemical gradients, directional motion, swarming, etc.), driving a system away from equilibrium. Nature provides numerous examples at all scales, ranging from macroscopic flocking of birds and schooling of fish to microscopic swarming of catalytic colloids and even the directional movement of active enzymes.^1–6^

Among these systems, enzymes are attractive candidates for studying out-of-equilibrium behavior at the nanoscale: they harvest energy from the environment, catalyzing chemical reactions to power motion.^7–9^ It has been demonstrated that enzymes can migrate toward/away from their substrate concentration gradients, a phenomenon called chemotaxis.^10–12^ When anchored onto larger microparticles, these particles also show chemotaxis.^13,14^ For multienzyme systems involving a catalytic cascade or a cycle, enzyme chemotaxis can further generate nonreciprocal interactions—a hallmark of active matter that seems to violate Newton’s third law of effective interactions.^15–17^ Understanding the mechanism behind enzyme chemotactic behavior gives us fundamental insights into the origin of life and information storage, and leads to important practical applications such as the creation of synthetic life-like systems and targeted drug delivery.

However, the governing principles of enzyme chemotaxis at the microscale and nanoscale are different. An active enzyme-functionalized microparticle can create local chemical gradients of either the substrate or the product, thereby breaking symmetry across their length scale. This induces directional motion via phoretic or hydrodynamic effects.^18,19^ In contrast, at the nano (molecular) scale, the rapid rotational Brownian diffusion makes it unlikely for a nanosized enzyme to sense chemical gradients across its own length.^20^ For a 10 nm size enzyme molecule, the time scale for its reorientation is approximately 700 ns $\left(\tau_{r}\approx \frac{1}{D_{r}}=\frac{8\pi \eta R^{3}}{k_{B}T}\right)$ . Recent studies have confirmed that directed motion and nonreciprocal interactions can emerge from the interplay between reaction kinetic asymmetry and the diffusion asymmetry between the free and substrate-bound states of the enzyme at the single-molecule level.^16,21^ Nevertheless, the dynamics underlying molecular chemotaxis are far more complex, as active enzymes can continuously reshape spatial and temporal chemical gradients through catalysis, thereby influencing the chemotactic behavior of neighboring enzyme molecules. To provide more practical insights for experimentalists and to advance the engineering of these systems, the dependence of molecular chemotactic velocity on catalyst concentration needs to be clearly elucidated.

Herein, we investigate the principles of active molecular chemotaxis using a Fokker–Planck model for an ATP-driven kinase-phosphatase system, explicitly focusing on the dependence of chemotactic velocity on enzyme concentration. The kinase-phosphatase enzyme pair regulates many cellular processes through an ATP-driven phosphorylation-dephosphorylation cycle.^22^ Using experimentally relevant enzyme concentrations (∼nM), we evaluate how energy supply, chemical gradients, and substrate binding govern molecular enzyme chemotaxis based on transient simulations. We track the evolution of chemical gradients, kinase concentration, free/bound enzyme ratio, and chemotactic velocity of the kinase ensemble. Furthermore, we provide nonequilibrium steady-state velocity maps that correlate chemotactic motion with energy input, chemical gradients, and enzyme concentrations.

Most notably, we demonstrate a nonmonotonic spatial chemotactic velocity profile, indicating a nonlinear interplay between reaction kinetics, enzyme conformational states (free vs bound state), and chemical gradients in the molecular system. This behavior is fundamentally distinct from microscale enzyme-functionalized particle chemotaxis, where diffusiophoretic chemotactic velocity typically scales linearly with the substrate concentration gradient (*V*_chemotaxis_ ∝ ∇*C*_substrate_).^17,18^ Intriguingly, similar nonmonotonic velocity profiles have also been reported in living bacteria,^23–25^ which arise when temporal signals overwhelm their spatial gradient processing capacity.^23^ While the sensing mechanism differs (a signal-processing network in bacteria versus physicochemical coupling in active enzymes), as we show, active enzymes can also exhibit nonmonotonic collective drift velocity based on spatial gradient alone. This work offers a detailed understanding of the factors affecting active molecular chemotaxis, providing valuable insights for the optimization of chemotactic performance.

## Results and Discussion

### Model System and Reaction Framework

To study kinase-phosphatase interaction and enzyme motion, we model a one-dimensional (1D) 20 μm microchannel in which kinase diffuses freely in the bulk, while phosphatase is immobilized on the left wall (Figure 1a). Driven by ATP hydrolysis, kinase (Kin) converts dephosphorylated protein (G) to phosphorylated protein (GP), while phosphatase (Pho) performs the opposite reaction (GP→*G*). Together, they form a cyclic reaction network (Figure 1b). Initially, kinase is uniformly distributed in the channel, and a constant supply of the substrate protein ([*G*_0_]) is set for the model. Upon the addition of ATP, kinase produces GP in the bulk, which then diffuses to the immobilized phosphatase wall and is converted back to *G*. Therefore, opposite spatial chemical gradients of [*G*] and [GP] emanate from the left wall (as shown in Figure 1a). As a result, kinase undergoes chemotaxis, as discussed below.

**Figure 1:**
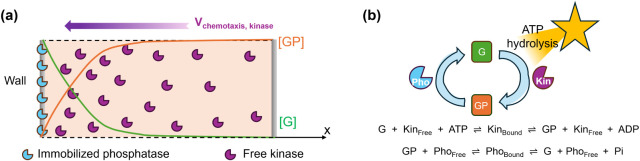
(a) Schematic of a microchannel with free kinase (Kin) in the bulk and immobilized phosphatase (Pho) on the left wall. Spatial chemical gradients are generated within the channel and induce chemotaxis of kinase. (b) Kinase catalyzes the conversion of dephosphorylated protein (*G*) into phosphorylated protein (GP), while phosphatase facilitates the reverse reaction (GP → *G*). Energy from adenosine triphosphate (ATP) hydrolysis can maintain the system away from thermodynamic equilibrium (enzyme size and quantity are not to scale in the diagram).

The top, bottom, and right boundaries of the channel are treated as semipermeable membranes, allowing small molecules (ATP, adenosine diphosphate (ADP), and inorganic phosphate (Pi)) to pass through. These small molecules’ concentrations (*C*_ATP_, *C*_ADP_, *C*_Pi_) are assumed to be constant during simulation, as living cells usually continuously replenish their ATP pool to maintain a stable energy charge and to fuel metabolic reactions.^26^

To study kinase chemotaxis, we employ the Fokker–Planck equation, a powerful framework for modeling the probability distribution of an enzyme ensemble under the influence of both thermal fluctuations and deterministic forces. We begin with reaction-diffusion models and transform the governing equation for total kinase concentration into the Fokker–Planck form (see the Methods section for the derivation process and model solving):

1 $$\frac{\partial {[\mathrm{Kin}]}_{T}}{\partial t}=\frac{\partial }{\partial x}\left(D\left(x,t\right)\frac{\partial {[\mathrm{Kin}]}_{T}\left(x,t\right)}{\partial x}-V\left(x,t\right){[\mathrm{Kin}]}_{T}\left(x,t\right)\right)$$

2 $$\delta_{\mathrm{Kin}}=\frac{D_{Kin_{B}}}{D_{Kin_{F}}},\;D\left(x,t\right)=D_{Kin_{F}}\frac{\left(1+\delta_{\mathrm{Kin}}\lambda \left(x,t\right)\right)}{1+\lambda \left(x,t\right)},\;V\left(x,t\right)=D_{Kin_{F}}\frac{\left(1-\delta_{\mathrm{Kin}}\right)}{{\left(1+\lambda \left(x,t\right)\right)}^{2}}\frac{\partial \lambda \left(x,t\right)}{\partial x}$$

3 $$\lambda \left(x,t\right)=\frac{{[\mathrm{Kin}]}_{B}}{{[\mathrm{Kin}]}_{F}}=\frac{k_{\mathrm{on},G}^{\mathrm{Kin}}C_{\mathrm{ATP}}\left[G\right]+k_{\mathrm{on},\mathrm{GP}}^{\mathrm{Kin}}C_{\mathrm{ADP}}\left[\mathrm{GP}\right]}{k_{\mathrm{off},G}^{\mathrm{Kin}}+k_{\mathrm{off},\mathrm{GP}}^{\mathrm{Kin}}}$$

eq 1 is the Fokker–Planck equation used to study the kinase system’s behavior. [Kin]_T_ represents the total kinase concentration (free state + substrate-bound state) at different positions *x* and times *t*, and *δ*_Kin_ is the ratio of the diffusion coefficient of bound $\left(D_{Kin_{B}}\right)$ and free kinase $\left(D_{Kin_{F}}\right)$ . The size, and hence the diffusivity, of an enzyme can vary depending on whether it is in the free or bound state.^21,27–29^ *D*(*x*,*t*) is the effective kinase diffusion coefficient, *V*(*x*,*t*) is the kinase ensemble chemotactic drift velocity, and *λ*(*x*,*t*) is the ratio of the concentration of bound ([Kin]_B_) and free (Kin]_F_) kinase, further controlled by kinetic asymmetry. Kinetic asymmetry can be interpreted as a difference in the transition state energies for the dissociation/association of *G* and GP.^21^ In the presence of a gradient of its substrate, an enzyme will move in the direction where the most diffusive conformational state is favored by the kinetic asymmetry, and the drift velocity is a function of the kinetic asymmetry, as shown in eq 2.^21,27–29^ *C*_ATP_, *C*_ADP_, *C*_Pi_ are fixed ATP, ADP, and Pi concentrations. $k_{i,j}^{e}$ is the rate constant for a specific reaction step (*i*: on, forward; off, backward, *j*: *G* or GP, *e*: Kin or Pho), which is defined as follows:

$$G+Kin_{\mathrm{Free}}+\mathrm{ATP}\overset{k_{\mathrm{on},G}^{\mathrm{Kin}}}{\underset{k_{\mathrm{off},G}^{\mathrm{Kin}}}{⇌}}Kin_{\mathrm{Bound}}\overset{k_{\mathrm{off},\mathrm{GP}}^{\mathrm{Kin}}}{\underset{k_{\mathrm{on},\mathrm{GP}}^{\mathrm{Kin}}}{⇌}}\mathrm{GP}+Kin_{\mathrm{Free}}+\mathrm{ADP}$$ 4

5 $$\mathrm{GP}+Pho_{\mathrm{Free}}\overset{k_{\mathrm{on},\mathrm{GP}}^{\mathrm{Pho}}}{\underset{k_{\mathrm{off},\mathrm{GP}}^{\mathrm{Pho}}}{⇌}}Pho_{\mathrm{Bound}}\overset{k_{\mathrm{off},G}^{\mathrm{Pho}}}{\underset{k_{\mathrm{on},G}^{\mathrm{Pho}}}{⇌}}G+Pho_{\mathrm{Free}}+\mathrm{Pi}$$

### System Evolution Driven by Energy Input

To investigate how energy input drives the system out of equilibrium and powers kinase chemotactic motion, we perform a transient simulation to capture system’s evolution from an initial homogeneous state to a final nonequilibrium steady state (NESS) (Figure 2). As described by eq 1, the obtained NESS spatial chemotactic velocity profile reflects the balance between diffusive flux and chemotactic flux. Initially (time = 0 s), all species ([*G*], [Kin]_T_) are set to be uniformly distributed in the microchannel, and the kinase chemotactic velocity is zero (Figure 2a-c, blue curves/symbols). The concentrations of ATP, ADP, and Pi are set such that the reaction quotient of ATP hydrolysis $(Q_{\mathrm{ATP}}=\frac{C_{\mathrm{ADP}}\times C_{\mathrm{Pi}}}{C_{\mathrm{ATP}}\times C^{o}}$ , where concentrations are normalized by the standard state concentration *C* ^o^) equals the reported equilibrium constant (*K*_ATP,eq_).^30^ Consequently, there is no free energy supply from ATP hydrolysis $(\Delta \mu_{\mathrm{ATP}}=\mu_{\mathrm{ATP}}-\mu_{\mathrm{ADP}}-\mu_{\mathrm{Pi}}=k_{B}T\times \ln\left(\frac{K_{\mathrm{ATP},\mathrm{eq}}}{Q_{\mathrm{ATP}}}\right)=0$ , see Methods section for more details). As time increases, substrate *G* and product GP concentration gradients emerge due to the cyclic reaction between bulk kinase and left-wall immobilized phosphatase. Without an additional external free energy source from ATP hydrolysis, the system evolves to a thermodynamic equilibrium state after sufficient time (Figure 2a-c, yellow curves). The resulting kinase concentration and chemotactic velocity profiles are compared with those in the initial homogeneous state (Figure 2b-c). Under thermodynamic equilibrium conditions, the kinase concentration remains uniform, and no chemotactic motion occurs.

**Figure 2:**
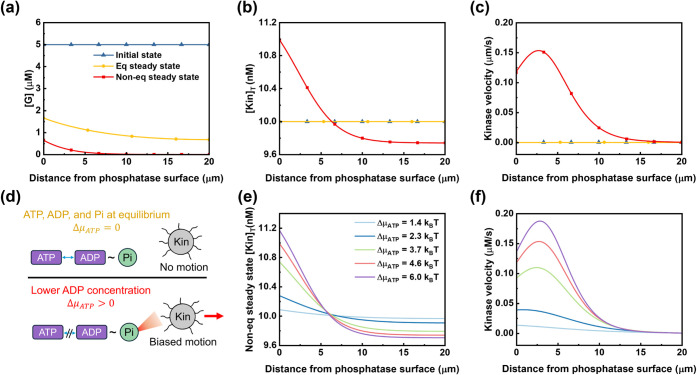
System evolution driven by ATP hydrolysis. The simulation initially runs at a thermodynamic equilibrium steady state (Δμ_ATP_ = 0) and is subsequently driven into a nonequilibrium steady state (NESS, Δμ_ATP_ > 0) by lowering the ADP concentration. Comparison of the (a) substrate concentration [*G*], (b) total kinase concentration [Kin]_T_, and (c) chemotactic velocity profiles of the kinase ensemble for the initial homogeneous state, the thermodynamic equilibrium steady state, and the final NESS (conducted with C_ADP_ = 4 × 10³ μM). Positive chemotactic velocity is defined as motion directed toward the immobilized phosphatase surface. The same legend is used for (a–c). For clarity, different symbols are added to distinguish the positions of overlapping curves. (d) Schematic diagram showing how lowering the ADP concentration drives the system away from equilibrium. (e) Total kinase concentration and (f) chemotactic velocity profiles at NESS under different energy inputs. Different Δμ_ATP_ values are achieved by altering C_ADP_ (10^5^, 4 × 10^4^, 10^4^, 4 × 10^3^, and 10^3^ μM). The same legend applies to panels (e,f). For the thermodynamic equilibrium state simulation, the reaction quotient of ATP hydrolysis is set to be equal to its equilibrium constant $\left(Q_{\mathrm{ATP}}=\frac{C_{\mathrm{ADP}}\times C_{\mathrm{Pi}}}{C_{\mathrm{ATP}}\times C^{o}}=K_{\mathrm{ATP},\mathrm{eq}}\right)$ . The system transitions to NESS by lowering C_ADP_, generating a chemical potential change $\left(\Delta \mu_{\mathrm{ATP}}=k_{B}T\times \ln\left(\frac{K_{\mathrm{ATP},\mathrm{eq}}}{Q_{\mathrm{ATP}}}\right)\right)$ . Initial conditions: [*G*_0_] = 5 μM, [GP_0_] = 0 μM, [Kin]_T,*t*0_ = 10 nM, [Pho]_T,*t*0_ = 0.2 nmol·m^‑2^. Detailed kinetic and diffusion parameters are listed in Table S1.

To drive the system out of equilibrium, we lower the concentration of ADP, leading to nonzero free energy change for ATP hydrolysis (Δμ_ATP_ >0) and providing a continuous energy source for the system (Figure 2d). After sufficient time, the system reaches the NESS. More kinases accumulate near the immobilized phosphatase surface (Figure 2b, red curve), and a nonzero chemotactic velocity profile develops (Figure 2c, red curve). Throughout our discussion, we define positive chemotactic velocity as motion directed toward the immobilized phosphatase surface. Since kinase’s substrate [*G*] concentration gradient is generated from the left immobilized phosphatase surface, we refer to this gradient-following motion as positive chemotaxis. Depending on kinetic and diffusion asymmetries,^21^ the kinase can also exhibit negative chemotaxis, moving away from the substrate *G* gradient and the immobilized phosphatase surface (Figure S1).

The time scale for establishing the gradient and reaching the nonequilibrium steady state is governed by the maximum reaction rate value (V_max_) of the enzyme and therefore can be adjusted by the proper choice of enzymes. For our simulations, we used parameters typical of fast enzymes, and the time scale is in seconds.

The term Δμ_ATP_ represents the free energy released from ATP hydrolysis at constant ATP, ADP, and Pi concentrations. To explore the relationship between the magnitude of this energy input into the system and the system’s chemotactic performance, we simulate the system at several different fixed ADP concentrations, calculate the corresponding energy, and show the corresponding NESS profiles. As the free energy released from ATP hydrolysis increases, both the accumulation of kinase near the wall (Figure 2e) and the peak chemotactic velocity in the channel increase (Figure 2f). This clearly shows the correlation between the energy supplied and the chemotactic performance of enzyme molecules.

### Chemotactic Velocity Behavior under Different Substrate Concentrations

An interesting observation from our simulations is the nonmonotonic chemotactic velocity profile in Figure 2f. Living bacteria exhibit similar nonmonotonic behavior when temporal chemical gradients overwhelm spatial cues, as they seek regions with the highest nutrient levels.^23^ Although the mechanisms differ, active enzymes can also exhibit nonmonotonic collective drift velocity due to a nonlinear interplay between reaction kinetics, enzyme conformational states (free vs bound state), and chemical gradients.

To better understand the origin of the nonmonotonic profile at the molecular level, we investigate system’s evolution with different initial substrate concentrations [*G*_0_] and obtain NESS profiles (Figure 3). As [*G*_0_] increases from 0.5 μM to 5 μM, the [*G*] gradient (slope of its concentration profile) becomes steeper and extends further into the bulk region at NESS (Figure 3a). Concomitantly, a greater local concentration shift of kinase toward the left immobilized phosphatase surface is observed (Figure 3b). However, both the peak chemotactic velocity and the chemotactic velocity profile shape do not change monotonically with respect to *G*_0_ concentration. As shown, the maximum local chemotactic velocity first increases as [*G*_0_] rises from 0.5 μM and then decreases at the higher [*G*_0_] supply concentration ([*G*_0_] = 5 μM) (Figure 3c). Correspondingly, the chemotactic velocity profile shape also changes from monotonic to nonmonotonic (Figure 3c, purple curve).

**Figure 3:**
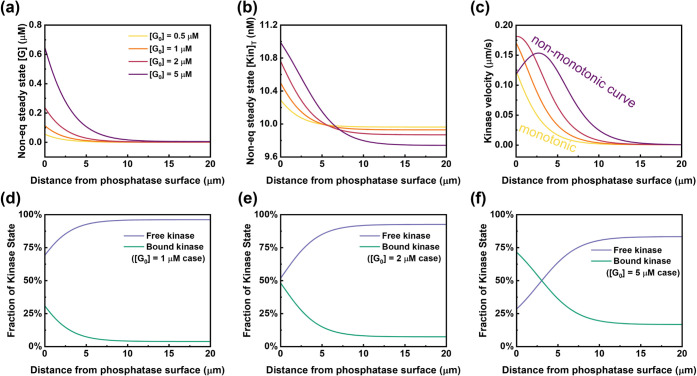
NESS system behavior at different initial substrate concentrations [*G*_0_]. (a) Substrate [*G*], (b) total kinase [Kin]_T_, and (c) kinase velocity profiles for initial substrate concentrations ([*G*_0_]) ranging from 0.5 to 5 μM. The same legend is used for (a–c). (d–f) NESS spatial profiles of the fraction of free and substrate-bound kinase for [*G*_0_] = 1 μM, 2 μM, and 5 μM cases, respectively. Δμ_ATP_ is set to 4.6 k_B_T. All other system parameters are held constant, and simulations are run until all profiles stabilize.

We hypothesize that the observed nonmonotonic velocity profile originates from the balance between enzyme’s free and substrate-bound states. An effective chemotactic drift requires a dynamic balance between the free and substrate-bound states of the enzymes, as no net chemotactic movement will occur if the enzyme population is either entirely free or completely saturated with the substrate. To test our hypothesis, we evaluate the fractions of free and substrate-bound kinase at NESS for different substrate concentrations ([*G*_0_]) (Figure 3d-f). As [*G*_0_] increases from 1 μM to 5 μM, the equilibrium enzyme population shifts toward the substrate-bound state. This is consistent with the deeper substrate gradients and stronger local enzyme accumulation observed previously at higher [*G*_0_] (Figure 3a-b). Crucially, the spatial distribution of these states does influence the shape of the chemotactic velocity profile. At a lower initial concentration ([*G*_0_] = 2 μM), the position where the free and bound fractions are approximately equal (a 50/50 balance) occurs at the left immobilized phosphatase surface. However, at a higher concentration ([*G*_0_] = 5 μM), this balance point shifts away from the phosphatase surface and into the bulk system. This shift corresponds directly to the emergence of a nonmonotonic velocity profile, with the peak velocity occurring near this balance point. Consequently, a decay in chemotactic velocity near the immobilized surface is observed because the local ratio of bound to free enzyme deviates from 1 (shifting toward saturation, Figure 3f). With further increases in [*G*_0_], the nonmonotonic velocity profile persists, and the peak velocity position shifts farther away from the left immobilized phosphatase surface (Figure S2), until the enzyme population transitions to the fully bound state, resulting in zero net chemotactic movement. The 50/50 balance point has a clear physical interpretation based on the enzyme’s binding affinity. The dissociation constant (*K*_D_) is, by definition, the substrate concentration at which half of the enzyme is in the bound state. It represents the point of maximum system sensitivity, in which the enzyme population has the greatest capacity to respond to both an increase and a decrease in [*G*]. Therefore, our results show that the spatial distribution of the enzyme’s free and substrate-bound states has a substantial impact on both the peak chemotactic velocity and the overall velocity profile shape. In a recent theoretical study, Fukuda et al. also noted that the influence of intermediate states during a reaction cycle on motor performance remains largely unexplored and showed that motor kinetics can be enhanced by optimizing intermediate-state properties.^31^

Building on the above analysis, we next examine how the nonmonotonic chemotactic velocity correlates with local substrate [*G*] and enzyme [Kin]_T_ concentration gradients, using data from the [*G*_0_] = 5 μM case (Figure 4a). It clearly underscores that, at the molecular level, kinase chemotactic velocity is proportional to the kinase concentration gradient, rather than the substrate [*G*] gradient. The linear fitting between velocity and d­[*G*]/d*x* or d­[Kin]_T_/d*x* further supports our observations (Figure 4b,c). Kinase chemotactic velocity is proportional to its concentration gradient (*V*_chemotaxis_ ∝ ∇[Kin]_T_), with an *R* ^2^ value of 0.996. This linear correlation can be explained as follows: the position of the maximum enzyme chemotactic drift/flux plane corresponds to the position where the difference in the enzyme’s concentration between its “two sides” is greatest. In other words, this reflects the position of the steepest gradient $\left(\frac{d{[\mathrm{Kin}]}_{T}}{dx}{|}_{\max}\right)$ in enzyme concentration, which is also the curve’s inflection point $\left(\frac{d^{2}{[\mathrm{Kin}]}_{T}}{dx^{2}}=0\right)$ . While the substrate and product concentration gradients determine the enzyme concentration gradient and free/bound state ratio, they do not directly determine its chemotactic velocity. In addition, although a substrate concentration gradient is necessary for molecular-level chemotaxis to occur, the substrate gradient alone is not sufficient to predict the direction of chemotaxis or the magnitude of chemotactic velocity. Thus, because of the different governing mechanisms, a direct analogy between the chemotaxis of nanoscale enzyme molecules and microscale enzyme-attached particles is not appropriate. Care should be taken to distinguish between these two length scales to avoid confusion.

**Figure 4:**
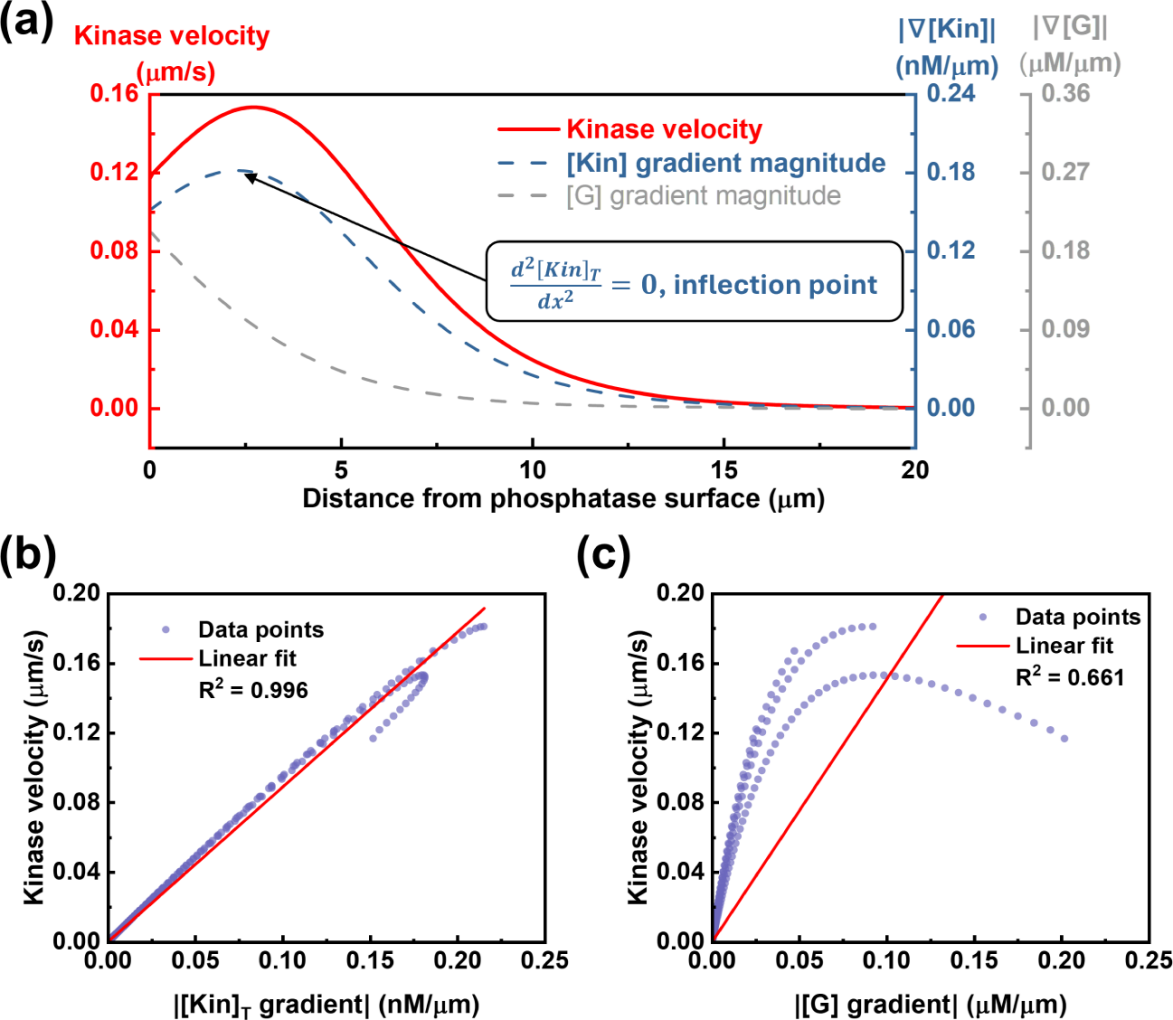
Analysis of chemotactic velocity dependence of active enzymes. (a) A comparison of the nonmonotonic kinase velocity profile (red solid line) with the enzyme (∇[Kin]_T_, blue dashed line) and substrate (∇[*G*], gray dashed line) gradients. The data shown are for an initial substrate concentration ([*G*_0_]) of 5 μM. (b) A plot of the local kinase velocity versus the local kinase concentration gradient reveals a strong linear correlation. (c) A plot of the local kinase velocity versus the local [*G*] concentration gradient shows a complex, nonlinear relationship. For panels (b) and (c), data points from various [*G*_0_] (= 0.5, 1, 2, 5 μM) conditions are aggregated to evaluate the global correlations. The observed multivalued trend is a direct result of the nonmonotonic velocity profiles. Specifically, a single velocity value in (a) can exist at two different positions in space, and these positions have different corresponding substrate/enzyme gradients, leading to the observed pattern. All other system parameters are held constant, and simulations are run until all profiles stabilize.

### Chemotactic Velocity Maps

To generalize our findings for active molecular chemotaxis, Figure 5 maps the NESS peak kinase chemotactic velocity (defined as the spatial maximum velocity under each condition) across a wide parameter space. These maps reveal how chemotaxis is coregulated by substrate concentration, energy supply, and enzyme concentration. In this system, bulk kinase consumes *G*, while immobilized phosphatase produces [*G*] from the left wall. To maintain a balanced catalytic cycle, we set the total number of surface phosphatase to be comparable to the total number of bulk kinase $\left(\frac{N_{A}\times {[\mathrm{Pho}]}_{T,\mathrm{surface}}\times \mathrm{Surface}\;\mathrm{area}}{N_{A}\times {[\mathrm{Kin}]}_{T,\mathrm{bulk}}\times L_{\mathrm{channel}}\times \mathrm{Surface}\;\mathrm{area}}\approx O\left(1\right)\right)$ .

**Figure 5:**
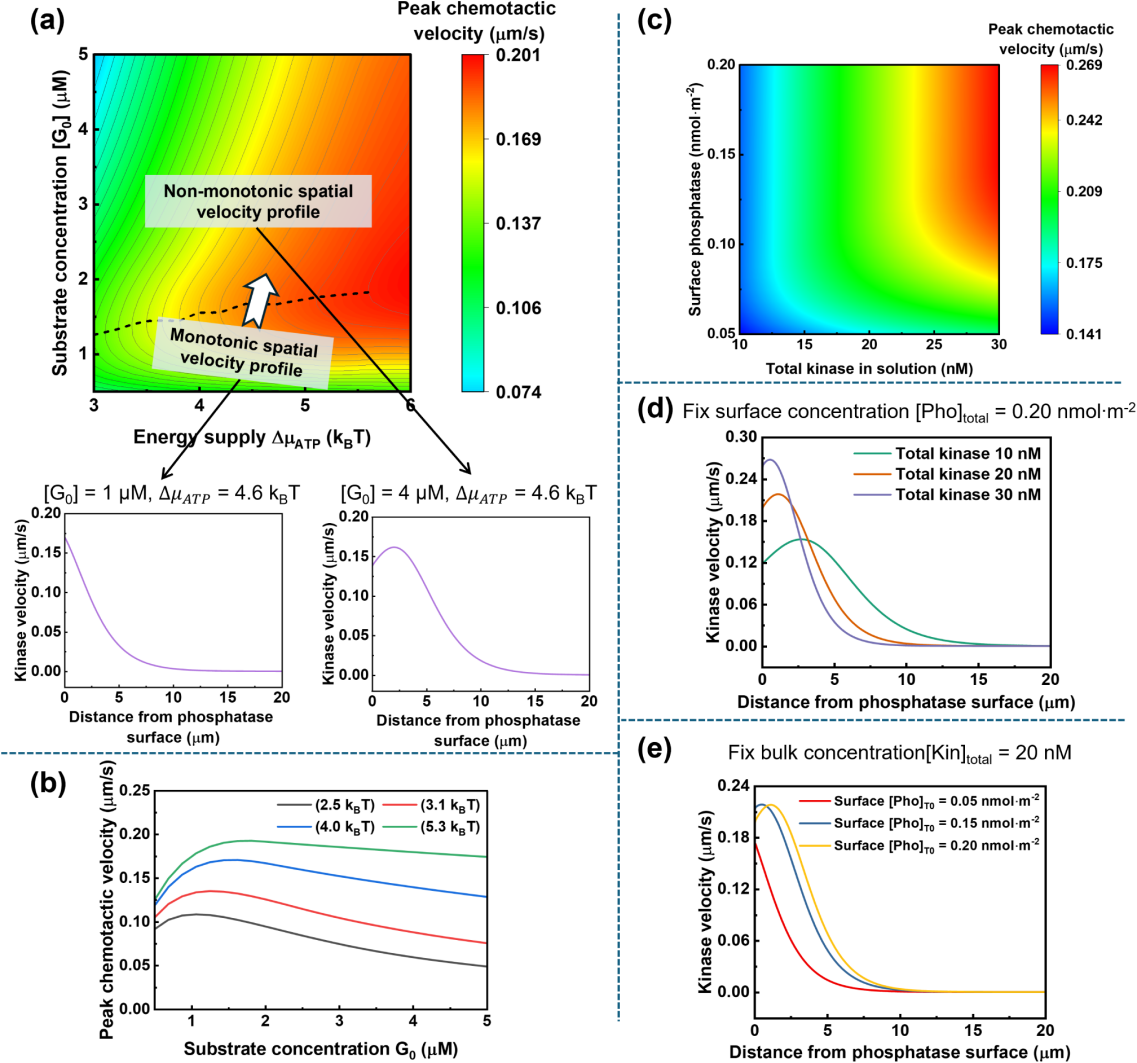
Mapping of NESS kinase chemotactic velocity across a wide parameter space. (a) NESS kinase peak chemotactic velocity (the spatial maximum velocity) as a function of energy supply (Δμ_ATP_) and initial substrate concentration. The gray contour lines connect points of equal peak chemotactic velocity. The dashed black line indicates the approximate threshold at which the system’s spatial velocity profile transitions from monotonic to nonmonotonic. The linked two plots below show the monotonic and nonmonotonic spatial velocity profiles under different conditions. For these simulations, the kinase concentration is held at 10 nM and the surface phosphatase concentration at 0.2 nmol·m^–2^. (b) A plot of the peak kinase chemotactic velocity versus the initial substrate concentration ([*G*_0_] = 0.5–5 μM) for different constant levels of energy supply (Δμ_ATP_ = 2.5–5.3 k_B_T). At a given energy supply, supplying substrate [*G*_0_] beyond a certain threshold reduces the peak chemotactic velocity. (c) NESS kinase peak chemotactic velocity as a function of total bulk kinase concentration and surface-immobilized phosphatase concentration. For these simulations, the initial substrate concentration is held constant at [*G*_0_] = 5 μM and the energy supply is Δμ_ATP_ = 4.6 k_B_T. (d) Spatial profiles of kinase velocity at a fixed surface phosphatase concentration ([Pho]_T,*t*0_ = 0.2 nmol·m^–2^) with varying bulk kinase concentrations (from 10 nM to 30 nM). (e) Spatial profiles of kinase velocity at a fixed bulk kinase concentration ([Kin]_T,*t*0_ = 20 nM) with varying surface phosphatase concentrations (from 0.05 nmol·m^–2^ to 0.2 nmol·m^–2^). All other system parameters are held constant, and simulations are run until all profiles stabilize.

Figure 5a shows the impact of energy supply (Δμ_ATP_) and initial substrate concentration ([*G*_0_]) on the peak kinase chemotactic velocity in the channel. While higher energy input consistently leads to higher chemotactic velocity, the relationship between velocity and initial substrate concentration is nonmonotonic. At a given energy supply (a fixed *x*-axis value), increasing the substrate concentration along the *y*-axis initially leads to a significant increase in the peak chemotactic velocity, which eventually saturates or even decreases. Based on our previous analysis, this behavior is related to the ratio of free to substrate-bound enzymes and the concentration gradient of the total enzyme (free state + bound state) population. Supplying substrate [*G*_0_] beyond a certain threshold reduces the peak chemotactic velocity (Figure 5b) and causes the spatial velocity profile shape to shift from monotonic to nonmonotonic (see linked two plots in Figure 5a). By examining the change in peak chemotactic velocity with respect to substrate concentration $\left(\frac{\partial v_{\mathrm{peak}}}{\partial \left[G_{0}\right]}\right)$ , we identify the threshold for this behavioral transition, indicated by the dashed black line in Figure 5a. Below this threshold, the peak velocity increases with initial substrate concentration [*G*_0_]. When [*G*_0_] is higher than this threshold, the peak velocity decreases, and a nonmonotonic spatial chemotactic velocity profile appears.

Figure 5c illustrates the role of enzyme concentration in regulating chemotaxis performance. Within a proper concentration range, increasing the concentration of either catalytic component (bulk kinase or immobilized phosphatase) leads to a higher kinase peak chemotactic velocity. Although both enhance chemotaxis, they operate via distinct mechanisms: higher bulk kinase concentration sharpens local chemical gradients, causing steeper concentration shifts during chemotaxis (Figure S3). In contrast, higher surface phosphatase concentration produces more [*G*], leading to a longer-range gradient and extending the interaction distance with the kinase in the bulk solution (Figure S4). Figure 5d and e further show detailed spatial chemotactic velocity profiles under different bulk kinase or immobilized phosphatase concentrations. With increasing enzyme concentration, higher local kinase velocities are observed near the catalytic phosphatase wall. Together, these findings suggest that the effective interaction between kinase and phosphatase can be tuned by varying their concentrations. Precise control of this effective interaction will be essential for engineering chemotaxis-mediated collective behavior, including metabolon formation and nonreciprocal phase transitions.

## Conclusions

In this work, we have investigated the principles governing active molecular chemotaxis and its velocity response within a dynamic, nonequilibrium environment. By using a kinase-phosphatase cyclic reaction system and immobilizing one enzyme component on the wall, chemical gradients are self-generated in the system and dynamically regulated by enzyme concentration and kinetics. We show that higher energy supply from ATP hydrolysis leads to increased chemotactic velocity and greater enzyme accumulation at the nonequilibrium steady state. We observed that the spatial chemotactic velocity profile transitions from monotonic to nonmonotonic curves as substrate concentration increases. Notably, at the molecular level, chemotactic velocity is governed by the interplay between the enzyme’s own concentration gradient, its free/bound state ratio, and chemical gradients. While substrate and product gradients are required to initiate chemotaxis, they do not solely determine the directionality or the chemotactic velocity profile. The chemotactic velocity maps further illustrate how energy supply, substrate concentration, and enzyme concentration modulate kinase chemotactic behavior. Furthermore, our results suggest that the effective interaction strength between kinase and phosphatase is tunable via enzyme concentration, and this tunability is crucial for regulating chemotaxis-mediated collective behaviors.

Energy supply, spatial chemical gradients, and enzyme states (free and bound) are three key components controlling the chemotactic performance of enzymes. Our findings highlight the fundamental differences between micro- and nanoscale chemotaxis. Unlike microscale objects, which can sense gradients across their body and migrate via diffusiophoretic motion, nanoscale enzymes rely on a combination of diffusion and kinetic asymmetry to achieve directional motion. Beyond clarifying these distinctions, our work also offers valuable insights into molecular system sensing and navigation. Looking forward, although some challenges still need to be addressed for experimental implementation (e.g., precise control of gradient formation, high-resolution imaging, and accurate single-molecule tracking), this work establishes a theoretical basis for future experimental platforms aimed at designing advanced active molecular chemotactic systems.

## Methods

We developed reaction-diffusion models for the kinase-phosphatase system. Based on reaction equations of eqs 4 and 5, the mass transfer equations for free [Kin]_F_ and bound ([Kin]_B_) kinase in the 1D microchannel can be expressed as follows:

6 $$\frac{\partial {[\mathrm{Kin}]}_{F}}{\partial t}=D_{Kin_{F}}\frac{\partial^{2}{[\mathrm{Kin}]}_{F}}{\partial x^{2}}-k_{\mathrm{on},G}^{\mathrm{Kin}}C_{\mathrm{ATP}}{[\mathrm{Kin}]}_{F}\left[G\right]-k_{\mathrm{on},\mathrm{GP}}^{\mathrm{Kin}}C_{\mathrm{ADP}}{[\mathrm{Kin}]}_{F}\left[\mathrm{GP}\right]+\left(k_{\mathrm{off},G}^{\mathrm{Kin}}+k_{\mathrm{off},\mathrm{GP}}^{\mathrm{Kin}}\right){[\mathrm{Kin}]}_{B}$$

7 $$\frac{\partial {[\mathrm{Kin}]}_{B}}{\partial t}=D_{Kin_{B}}\frac{\partial^{2}{[\mathrm{Kin}]}_{B}}{\partial x^{2}}+k_{\mathrm{on},G}^{\mathrm{Kin}}C_{\mathrm{ATP}}{[\mathrm{Kin}]}_{F}\left[G\right]+k_{\mathrm{on},\mathrm{GP}}^{\mathrm{Kin}}C_{\mathrm{ADP}}{[\mathrm{Kin}]}_{F}\left[\mathrm{GP}\right]-\left(k_{\mathrm{off},G}^{\mathrm{Kin}}+k_{\mathrm{off},\mathrm{GP}}^{\mathrm{Kin}}\right){[\mathrm{Kin}]}_{B}$$

Given that chemical transformations are much faster than the diffusion of the proteins,^16^ if we applied the fast chemistry assumption for kinase, one can obtain

8 $$0=-k_{\mathrm{on},G}^{\mathrm{Kin}}C_{\mathrm{ATP}}{[\mathrm{Kin}]}_{F}\left[G\right]-k_{\mathrm{on},\mathrm{GP}}^{\mathrm{Kin}}C_{\mathrm{ADP}}{[\mathrm{Kin}]}_{F}\left[\mathrm{GP}\right]+\left(k_{\mathrm{off},G}^{\mathrm{Kin}}+k_{\mathrm{off},\mathrm{GP}}^{\mathrm{Kin}}\right){[\mathrm{Kin}]}_{B}$$

The ratio between bound and free form of kinase can be further expressed as

9 $$\lambda \left(x,t\right)=\frac{{[\mathrm{Kin}]}_{B}}{{[\mathrm{Kin}]}_{F}}=\frac{k_{\mathrm{on},G}^{\mathrm{Kin}}C_{\mathrm{ATP}}\left[G\right]+k_{\mathrm{on},\mathrm{GP}}^{\mathrm{Kin}}C_{\mathrm{ADP}}\left[\mathrm{GP}\right]}{k_{\mathrm{off},G}^{\mathrm{Kin}}+k_{\mathrm{off},\mathrm{GP}}^{\mathrm{Kin}}}$$

Total kinase concentration ([Kin]_T_) is

10 $${[\mathrm{Kin}]}_{T}\left(x,t\right)={[\mathrm{Kin}]}_{F}\left(x,t\right)+{[\mathrm{Kin}]}_{B}\left(x,t\right)$$

11 $${[\mathrm{Kin}]}_{F}\left(x,t\right)=\frac{{[\mathrm{Kin}]}_{T}\left(x,t\right)}{1+\lambda \left(x,t\right)},{[\mathrm{Kin}]}_{B}\left(x,t\right)=\frac{{[\mathrm{Kin}]}_{T}\left(x,t\right)}{1+\lambda \left(x,t\right)}\lambda \left(x,t\right)$$

By combining eqs 6, 7, 10 and 11 together, we derived the Fokker-Planck form equation for the total kinase concentration as follows (see full derivation in Supporting Information):

12 $$\begin{array}{ll} \frac{\partial {[\mathrm{Kin}]}_{T}}{\partial t} & =\frac{\partial {[\mathrm{Kin}]}_{F}}{\partial t}+\frac{\partial {[\mathrm{Kin}]}_{B}}{\partial t}=D_{Kin_{F}}\frac{\partial^{2}{[\mathrm{Kin}]}_{F}}{\partial x^{2}}+D_{Kin_{B}}\frac{\partial^{2}{[\mathrm{Kin}]}_{B}}{\partial x^{2}}\\ & =D_{Kin_{F}}\frac{\partial^{2}\left(\frac{{[\mathrm{Kin}]}_{T}\left(x,t\right)}{1+\lambda \left(x,t\right)}\right)}{\partial x^{2}}+\delta_{\mathrm{Kin}}D_{Kin_{F}}\frac{\partial^{2}\left(\frac{{[\mathrm{Kin}]}_{T}\left(x,t\right)}{1+\lambda \left(x,t\right)}\lambda \left(x,t\right)\right)}{\partial x^{2}}\\ & \to \frac{\partial {[\mathrm{Kin}]}_{T}}{\partial t}=\frac{\partial }{\partial x}\left(D\left(x,t\right)\frac{\partial {[\mathrm{Kin}]}_{T}\left(x,t\right)}{\partial x}-V\left(x,t\right){[\mathrm{Kin}]}_{T}\left(x,t\right)\right) \end{array}$$

13 $$\delta_{\mathrm{Kin}}=\frac{D_{Kin_{B}}}{D_{Kin_{F}}},D\left(x,t\right)=D_{Kin_{F}}\frac{\left(1+\delta_{\mathrm{Kin}}\lambda \left(x,t\right)\right)}{1+\lambda \left(x,t\right)},V\left(x,t\right)=D_{Kin_{F}}\frac{\left(1-\delta_{\mathrm{Kin}}\right)}{{\left(1+\lambda \left(x,t\right)\right)}^{2}}\frac{\partial \lambda \left(x,t\right)}{\partial x}$$

eq 12 is the Fokker-Planck form equation for kinase ensemble transport, with defined parameters in eqs 9 and 13 together indicating that the chemotactic velocity direction is governed by the interplay of reaction kinetics, the enzyme diffusivity difference between free and bound states, and the concentration gradients. In order to solve the partial differential equation (eq 12), we also needed to know [*G*] and [GP] spatial concentration profiles. The mass transport equations of [*G*] and [GP] can be written as

14 $$\frac{\partial \left[G\right]\left(x,t\right)}{\partial t}=D_{G}\frac{\partial^{2}\left[G\right]\left(x,t\right)}{\partial x^{2}}-k_{\mathrm{on},G}^{\mathrm{Kin}}{[\mathrm{Kin}]}_{F}\left[G\right]\left[\mathrm{ATP}\right]+k_{\mathrm{off},G}^{\mathrm{Kin}}{[\mathrm{Kin}]}_{B}$$

$$\frac{\partial \left[\mathrm{GP}\right]\left(x,t\right)}{\partial t}=D_{\mathrm{GP}}\frac{\partial^{2}\left[\mathrm{GP}\right]\left(x,t\right)}{\partial x^{2}}-k_{\mathrm{on},\mathrm{GP}}^{\mathrm{Kin}}{[\mathrm{Kin}]}_{F}\left[\mathrm{GP}\right]\left[\mathrm{ADP}\right]+k_{\mathrm{off},\mathrm{GP}}^{\mathrm{Kin}}{[\mathrm{Kin}]}_{B}$$ 15

We assumed that the addition or removal of a phosphate group does not significantly affect the diffusion coefficient of a large protein molecule (*D*_G_ = *D*_GP_), and these equations were solved together to study molecular enzyme chemotaxis. All kinetic, diffusion, and system parameters are summarized in Table S1. The kinetic parameters used in the simulations were chosen to be within the order of magnitude of values reported in the literature.^32–38^ The partial differential equation—parabolic/elliptic (*PDEPE*) function in MATLAB—was used to solve one spatial dimension time-dependent PDEs here. Transient simulations were performed until all variable profiles reached a steady state and remained unchanged. No-flux boundary conditions were applied at the walls for the kinase, [*G*], and [GP]. Since the immobilizd [GP] and produced [*G*] as the reaction proceeded, the boundary conditions of [*G*] and [GP] at the left wall were set so that their diffusive flux matched the net local reaction flux, thereby maintaining mass balance at the reactive wall. In addition, during transient simulation, the actual net reaction fluxes for the equations associated with the fast chemistry assumption (e.g., eq 8) were continuously monitored. We set the convergence criterion as 10^–6^, maintaining a net reaction flux below this threshold for each time step throughout the transient simulation.

Free energy from ATP hydrolysis (Δμ_ATP_ = *μ*_ATP_ – *μ*_ADP_ – *μ*_Pi_) can be calculated based on ATP, ADP, and Pi concentrations, as well as the thermodynamic equilibrium constant of ATP hydrolysis.

16 $$\begin{array}{c} K_{\mathrm{ATP},\mathrm{eq}}=\frac{\frac{C_{\mathrm{ADP}}}{C^{o}}\times \frac{C_{\mathrm{Pi}}}{C^{o}}}{\frac{C_{\mathrm{ATP}}}{C^{o}}}{|}_{\mathrm{eq}}=\frac{C_{\mathrm{ADP}}\times C_{\mathrm{Pi}}}{C_{\mathrm{ATP}}\times C^{o}}{|}_{\mathrm{eq}}\\ \Delta \mu_{\mathrm{ATP}}=\Delta \mu_{\mathrm{ATP}}^{o}+k_{B}T\times \ln\left(\frac{C_{\mathrm{ATP}}\times C^{o}}{C_{\mathrm{ADP}}\times C_{\mathrm{Pi}}}\right)\\ \Delta \mu_{\mathrm{ATP}}=k_{B}T\times \ln\left(K_{\mathrm{ATP},\mathrm{eq}}\right)+k_{B}T\times \ln\left(\frac{C_{\mathrm{ATP}}\times C^{o}}{C_{\mathrm{ADP}}\times C_{\mathrm{Pi}}}\right)\\ \to \Delta \mu_{\mathrm{ATP}}=k_{B}T\times \ln\left(\frac{C_{ATP}\times C^{o}}{C_{\mathrm{ADP}}\times C_{\mathrm{Pi}}}\times K_{\mathrm{ATP},\mathrm{eq}}\right)=k_{B}T\times \ln\left(\frac{K_{\mathrm{ATP},\mathrm{eq}}}{Q_{\mathrm{ATP}}}\right) \end{array}$$

where *C*_ATP_, *C*_ADP_, *C*_Pi_ are ATP, ADP, and Pi concentrations used in the model. The equilibrium constant *K*_ATP,eq_ was written in its dimensionless activity form, where concentrations were normalized by the standard state concentration *C* ^o^ (1M). $\Delta \mu_{\mathrm{ATP}}^{o}$ is the change in chemical potential under thermodynamic equilibrium. *Q*_ATP_ is the reaction quotient of ATP hydrolysis. A reported equilibrium constant value, *K*_eq,reported_ = 2 × 10^5^, was used for equilibrium state concentration and chemical potential calculations.^30^

## Supplementary Material



## Notes

Derivation of the Fokker-Plank model from a reaction-diffusion model; demonstration of negative chemotaxis by tuning system diffusion or kinetic asymmetries; influence of various parameters (substrate concentration, kinase concentration and immobilized phosphatase concentration) on kinase chemotaxis performance; and summary of parameters used in the transient simulations (PDF)
