# Supplementary material for: Emergent Nonlinearity in Active Molecular Chemotaxis
Source: ACS Nano. 2026 May 13;20(20):14775–84. doi: 10.1021/acsnano.6c03464 (PMC13218049; doi:10.1021/acsnano.6c03464)
Supplement: Supplementary file 1 [file nn6c03464_si_001.pdf]

Supporting Information

Emergent Nonlinearity in Active Molecular Chemotaxis

Xiaotian Lu,[1] Ayusman Sen,[1,2]* and R. Dean Astumian[3]*

[1]Department of Chemical Engineering, The Pennsylvania State University, University Park, Pennsylvania 16802, United States

[2]Department of Chemistry, The Pennsylvania State University, University Park, Pennsylvania 16802, United States

[3]Department of Physics and Astronomy, University of Maine, Orono, ME 04469, United States

Corresponding Author E-mails: asen@psu.edu (AS), astumian@maine.edu (RDA)

1. Detailed derivation from a reaction-diffusion model to a Fokker-Plank model

$$\frac{\partial[Kin]_T}{\partial t} = \frac{\partial[Kin]_F}{\partial t} + \frac{\partial[Kin]_B}{\partial t}$$

$$= D_{Kin_F}\frac{\partial^2[Kin]_F}{\partial x^2} + D_{Kin_B}\frac{\partial^2[Kin]_B}{\partial x^2}$$

$$= D_{Kin_F}\frac{\partial^2(\frac{[Kin]_T(x,t)}{1+\lambda(x,t)})}{\partial x^2} + \delta_{Kin}D_{Kin_F}\frac{\partial^2(\frac{[Kin]_T(x,t)}{1+\lambda(x,t)}\lambda(x,t))}{\partial x^2}$$

$$= D_{Kin_F} * \frac{\partial\left(\frac{1}{1+\lambda(x,t)} * \frac{\partial[Kin]_T(x,t)}{\partial x} - \frac{[Kin]_T(x,t)}{\left(1+\lambda(x,t)\right)^2} * \frac{\partial\lambda(x,t)}{\partial x}\right)}{\partial x} + \delta_{Kin}D_{Kin_F}$$

$$* \frac{\partial\left(\frac{\lambda(x,t)}{1+\lambda(x,t)} * \frac{\partial[Kin]_T(x,t)}{\partial x} + \frac{[Kin]_T(x,t)}{\left(1+\lambda(x,t)\right)^2} * \frac{\partial\lambda(x,t)}{\partial x}\right)}{\partial x}$$

$$= \partial\left(\begin{matrix}\frac{D_{Kin_F}}{1+\lambda(x,t)} * \frac{\partial[Kin]_T(x,t)}{\partial x} - D_{Kin_F}\frac{[Kin]_T(x,t)}{\left(1+\lambda(x,t)\right)^2} * \frac{\partial\lambda(x,t)}{\partial x} + \\ \delta_{Kin}D_{Kin_F}\frac{\lambda(x,t)}{1+\lambda(x,t)} * \frac{\partial[Kin]_T(x,t)}{\partial x} + \delta_{Kin}D_{Kin_F} * \frac{[Kin]_T(x,t)}{\left(1+\lambda(x,t)\right)^2} * \frac{\partial\lambda(x,t)}{\partial x}\end{matrix}\right)/\partial x$$

$$= \frac{\partial\left(D_{Kin_F}\frac{1+\delta_{Kin} * \lambda(x,t)}{1+\lambda(x,t)} * \frac{\partial[Kin]_T(x,t)}{\partial x} - D_{Kin_F}\frac{[Kin]_T(x,t) * (1-\delta_{Kin})}{\left(1+\lambda(x,t)\right)^2} * \frac{\partial\lambda(x,t)}{\partial x}\right)}{\partial x}$$

$$= \frac{\partial}{\partial x}(D_{Kin_F}\frac{(1+\delta_{Kin}\lambda(x,t))}{1+\lambda(x,t)}\frac{\partial[Kin]_T(x,t)}{\partial x} - D_{Kin_F}\frac{(1-\delta_{Kin})}{\left(1+\lambda(x,t)\right)^2}\frac{\partial\lambda(x,t)}{\partial x}[Kin]_T(x,t))$$

$$\rightarrow \frac{\partial[Kin]_T}{\partial t} = \frac{\partial}{\partial x}(D(x,t)\frac{\partial[Kin]_T(x,t)}{\partial x} - V(x,t)[Kin]_T(x,t)) \quad \text{(S1)}$$

$$\delta_{Kin} = \frac{D_{Kin_B}}{D_{Kin_F}},\quad D(x,t) = D_{Kin_F}\frac{(1+\delta_{Kin}\lambda(x,t))}{1+\lambda(x,t)},\quad V(x,t) = D_{Kin_F}\frac{(1-\delta_{Kin})}{\left(1+\lambda(x,t)\right)^2}\frac{\partial\lambda(x,t)}{\partial x} \quad \text{(S2)}$$

$$\lambda(x,t) = \frac{[Kin]_B}{[Kin]_F} = \frac{k_{on,G}^{Kin}C_{ATP}[G] + k_{on,GP}^{Kin}C_{ADP}[GP]}{k_{off,G}^{Kin} + k_{off,GP}^{Kin}} \quad \text{(S3)}$$

Equation S1 is the Fokker-Planck equation to study the kinase system's behavior. Equations S2 and S3 are some defined parameters. $[Kin]_T$ is total kinase concentration (free state + bound state) at different position x and time t, δ_{Kin} is the ratio of diffusion coefficient of bound (D_{Kin_B}) and free kinase (D_{Kin_F}), $D(x,t)$ is effective kinase diffusion coefficient, $V(x,t)$ is the kinase ensemble chemotactic drift velocity, and $\lambda(x,t)$ is the ratio of concentration of

bound ($[Kin]_B$) and free ($[Kin]_F$) kinase. C_{ATP}, C_{ADP}, C_{Pi} are fixed ATP, ADP, and Pi concentrations. $k_{i,j}^{e}$ is rate constant for a specific reaction step shown in main manuscript (i: on, forward; off, backward, j: G or GP, e: Kin or Pho). Equations S2 and S3 demonstrate that the chemotactic velocity direction is governed by the interplay of reaction rate constants ($k_{i,j}^{e}$), the enzyme diffusivity difference between free and bound states, and the concentration gradients.

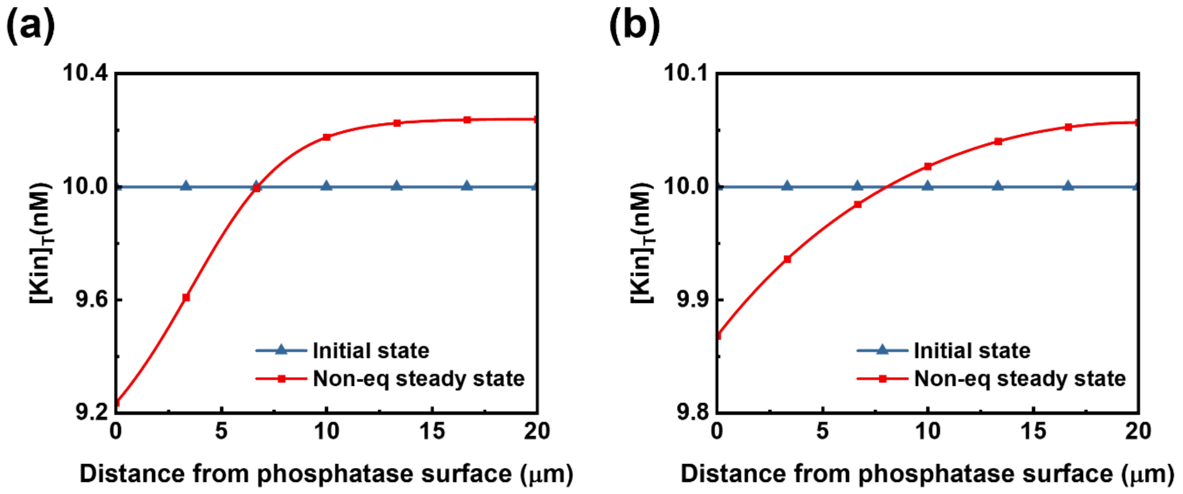

Figure S1. Demonstration of negative chemotaxis by tuning system diffusion or kinetic asymmetries. In contrast to the positive chemotaxis shown in **Figure 2**, negative chemotaxis is induced by modifying either (a) enzyme diffusion asymmetry ($\delta_{Kin} = \delta_{Pho} = 1.2$) or (b) reaction kinetic asymmetry ($k_{off,G}^{Kin} = 500\ s^{-1}, k_{off,GP}^{Kin} = 1\ s^{-1}, k_{on,GP}^{Pho} = 1\ \mu M^{-1}s^{-1}, k_{off,G}^{Pho} = 1\ s^{-1}$). Kinase moves away from the immobilized phosphatase surface, where its substrate concentration gradient is generated. All other simulation parameters are identical to those used in **Figure 2**.

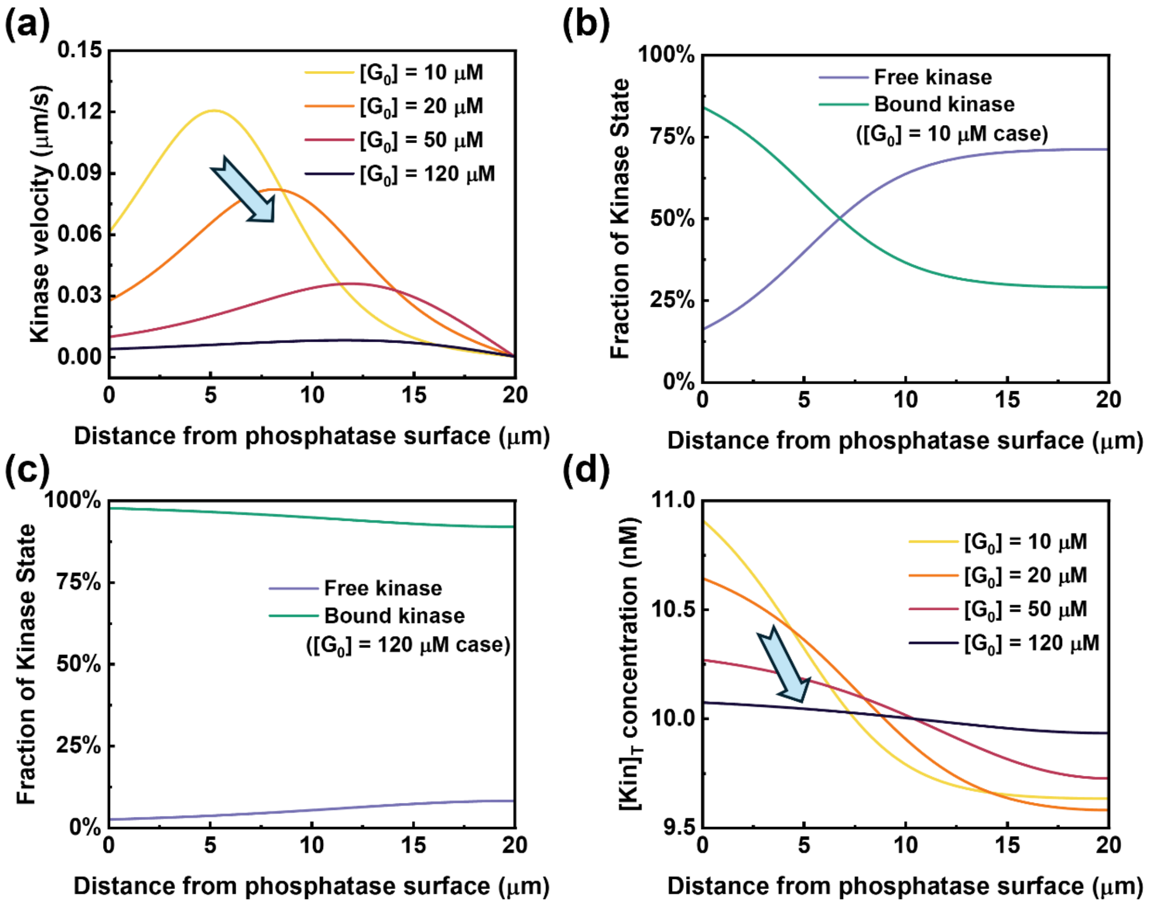

Figure S2. Non-equilibrium steady state system behavior at high initial substrate concentrations ($[G_0]$). This figure complements the main manuscript **Figure 3** by showing how the kinase's chemotactic response changes as enzyme population approaches substrate-bound state. (a) Kinase velocity profiles. (b) (c) Spatial profiles of the fraction of free and substrate-bound kinase for $[G_0]$ = 10 μM, 120 μM, respectively. (d) total kinase $[Kin]_T$ concentration profiles. All other system parameters are held constant, and simulations are run until all profiles stabilize. With further increases in $[G_0]$, the non-monotonic velocity profile shape persists, and the peak velocity position shifts farther away from the left phosphatase surface. Ultimately, the enzyme population becomes almost fully saturated and transitions to the substrate-bound state, causing the net chemotactic velocity/concentration shift to approach near-zero. Light blue arrows point to curves with higher $[G_0]$ concentration.

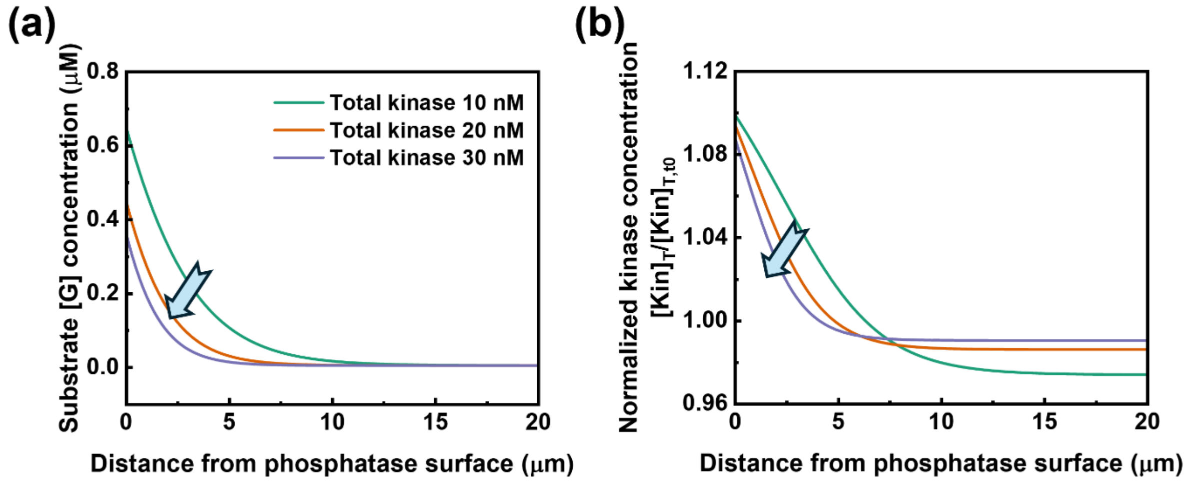

Figure S3. Non-equilibrium steady state system behavior at different total kinase concentrations. (a) Substrate [G], and (b) $[Kin]_T$ normalized by its initial concentration ($[Kin]_{T, t0}$) profiles for initial total kinase concentration ($[Kin]_{T, t0}$) ranging from 10 nM to 30 nM ($[Pho]_{T, t0}$ = 0.2 nmol·m^{-2}). All other simulation parameters are identical to those used in **Figure 5c and 5d**. Same legend is used for (a-b). Higher concentrations of kinase in the bulk solution can sharpen local G gradients, cause steeper concentration shifts during chemotaxis, and lead to higher local chemotaxis velocity. Light blue arrows point to curves with higher kinase concentration.

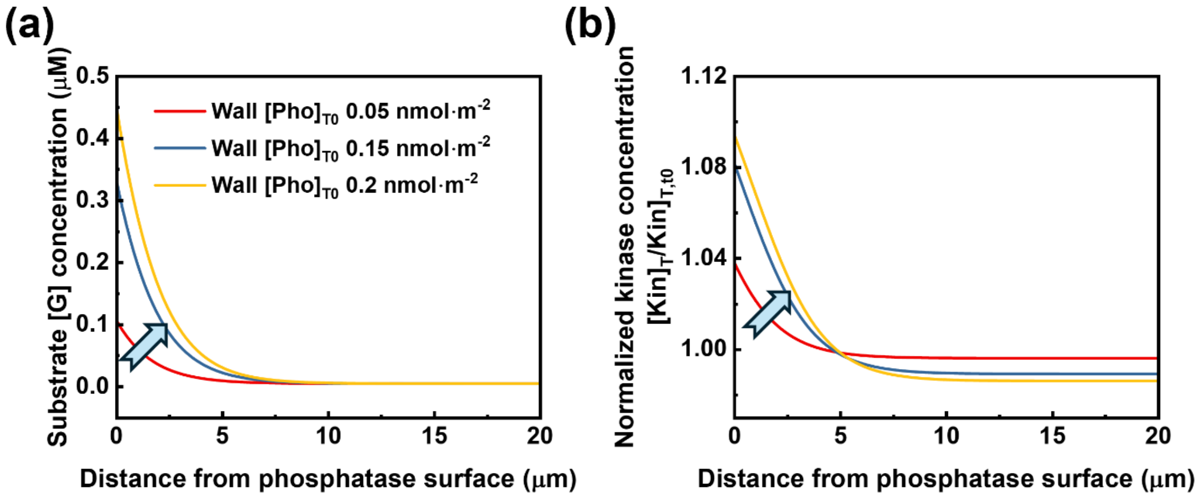

Figure S4. Non-equilibrium steady state system behavior at different initial surface phosphatase concentrations. (a) Substrate [G] and (b) $[Kin]_T$ normalized by its initial concentration ($[Kin]_{T,\ t0}$) profiles for initial surface phosphatase concentration ($[Pho]_{T,\ t0}$) ranging from 0.05 nmol·m^{-2} to 0.2 nmol·m^{-2} ($[Kin]_{T,\ t0}$ = 20 nM). All other simulation parameters are identical to those used in **Figure 5c and 5e**. Same legend is used for (a-b). Higher concentrations of surface phosphatase can produce more [G], leading to a longer-range gradient and extending interaction distance with bulk kinase. Light blue arrows point to curves with higher phosphatase concentration.

Table S1. Summary of parameters used in the transient simulations.

**Name**	**Parameters**	**Values**	**Unit**
Kinase (Kin): on rate constant for unphosphorylated G	$k_{on,G}^{Kin}$	200	$\mu M^{-2}s^{-1}$
Kin: off rate constant for unphosphorylated G	$k_{off,G}^{Kin}$	10	s^{-1}
Kin: on rate constant for phosphorylated GP	$k_{on,GP}^{Kin}$	10^{-3}	$\mu M^{-2}s^{-1}$
Kin: off rate constant for phosphorylated GP	$k_{off,GP}^{Kin}$	100	s^{-1}
Phosphatase (Pho): on rate constant for phosphorylated GP	$k_{on,GP}^{Pho}$	5	$\mu M^{-1}s^{-1}$
Pho: off rate constant for phosphorylated GP	$k_{off,GP}^{Pho}$	10	s^{-1}
Pho: on rate constant for unphosphorylated G	$k_{on,G}^{Pho}$	10^{-5}	$\mu M^{-2}s^{-1}$
Pho: off rate constant for unphosphorylated G	$k_{off,G}^{Pho}$	100	s^{-1}
Diffusion coefficient of free kinase	D_{Kin_F}	10	μm^2s^{-1}
Ratio of bound to free kinase diffusion coefficients	δ_{Kin}	0.8	-
Diffusion coefficient of free phosphatase	D_{Pho_F}	10	μm^2s^{-1}
Ratio of bound to free phosphatase diffusion coefficients	δ_{Pho}	0.8	-
Diffusion coefficient of unphosphorylated protein G	D_G	15	μm^2s^{-1}
Diffusion coefficient of phosphorylated protein GP	D_{GP}	15	μm^2s^{-1}
Constant ATP concentration	C_{ATP}	2	μM
Variable ADP concentration	C_{ADP}	10^3 to 4×10^5	μM
Constant Pi concentration	C_{Pi}	10^6	μM
Standard reference concentration	C^o	10^6	μM
ATP hydrolysis equilibrium constant	$K_{ATP,eq}$	2×10^5	-

Notes: Kinase and phosphatase reaction equations are shown in eqs 4 and 5. As multiple types of kinases[1–4] and phosphatases[5–7] exist in biological systems, their kinetics can vary substantially depending on the specific substrates (G and GP in this study) and the cellular

environment. The kinetic parameters used in the simulations were chosen to be within the order of magnitude of values reported in the above references.[1–7] The diffusion coefficient of protein kinase and phosphatase were set according to cytoplasmic conditions,[8] and diffusion coefficient of unphosphorylated G and phosphorylated GP proteins were chosen to be of the same order of magnitude. The equilibrium constant of ATP hydrolysis ($K_{ATP,eq}$) was calculated based on the reported Gibbs free energy.[9]

References

(1) Wang, C.; Lee, T. R.; Lawrence, D. S.; Adams, J. A. Rate-Determining Steps for Tyrosine Phosphorylation by the Kinase Domain of v-Fps. *Biochemistry* **1996**, *35*, 1533–1539.

(2) Adams, J. A. Kinetic and Catalytic Mechanisms of Protein Kinases. *Chem. Rev.* **2001**, *101*, 2271–2290.

(3) Salazar, C.; Höfer, T. Multisite Protein Phosphorylation – from Molecular Mechanisms to Kinetic Models. *FEBS J.* **2009**, *276*, 3177–3198.

(4) Søberg, K.; Skålhegg, B. S. The Molecular Basis for Specificity at the Level of the Protein Kinase a Catalytic Subunit. *Front. Endocrinol.* **2018**, *9*, 538.

(5) Zhang, Z. Y.; Maclean, D.; Thiemesefler, A. M.; Roeske, R. W.; Dixon, J. E. A Continuous Spectrophotometric and Fluorometric Assay for Protein Tyrosine Phosphatase Using Phosphotyrosine-Containing Peptides. *Anal. Biochem.* **1993**, *211*, 7–15.

(6) Parker, P. J.; Goris, J.; Merlevede, W. Specificity of Protein Phosphatases in the Dephosphorylation of Protein Kinase C. *Biochem. J.* **1986**, *240*, 63–67.

(7) Hertz, E. P. T.; Kruse, T.; Davey, N. E.; López-Méndez, B.; Sigurðsson, J. O.; Montoya, G.; Olsen, J. V.; Nilsson, J. A Conserved Motif Provides Binding Specificity to the PP2A-B56 Phosphatase. *Mol. Cell* **2016**, *63*, 686–695.

(8) Sherpa, R. T.; Moshal, K. S.; Agarwal, S. R.; Ostrom, R. S.; Harvey, R. D. Role of Protein Kinase A and A Kinase Anchoring Proteins in Buffering and Compartmentation of cAMP Signalling in Human Airway Smooth Muscle Cells. *Br. J. Pharmacol.* **2024**, *181*, 2622–2635.

(9) Guynn, R. W.; Veech, R. L. The Equilibrium Constants of the Adenosine Triphosphate Hydrolysis and the Adenosine Triphosphate-Citrate Lyase Reactions. *J. Biol. Chem.* **1973**, *248*, 6966–6972.
